# Antioxidantien bei eingeschränkter Zeugungsfähigkeit des Mannes

**DOI:** 10.1007/s00120-023-02184-4

**Published:** 2023-09-19

**Authors:** Sebastian Graf

**Affiliations:** 1 Klinik für Urologie und Andrologie, Kepler Universitätsklinikum Linz, Linz, Österreich; 2 UroEvidence@Deutsche Gesellschaft für Urologie, Martin-Buber-Str. 10, 14163 Berlin, Deutschland

## Originalpublikation

de Ligny W, Smits RM, Mackenzie-Proctor R et al (2022). Antioxidants for male subfertility. Cochrane Database of Systematic Reviews 2022, Issue 5. Art. No.: CD007411. 10.1002/14651858.CD007411.pub5.

## Hintergrund

Die Unfähigkeit, Kinder zu bekommen, betrifft 10–15 % der Paare weltweit. Man schätzt, dass bis zur Hälfte der Unfruchtbarkeitsfälle auf einen männlichen Faktor zurückzuführen sind, wobei man davon ausgeht, dass zwischen 25 und 87 % der männlichen Subfertilität auf die Auswirkungen von oxidativem Stress zurückzuführen sind. Es wird angenommen, dass die orale Einnahme von Antioxidantien die Spermienqualität verbessert, indem sie oxidative Schäden reduziert. Antioxidantien sind weithin verfügbar und im Vergleich zu anderen Fruchtbarkeitsbehandlungen kostengünstig. Allerdings unterliegen die meisten Antioxidantien keiner gesetzlichen Kontrolle, und die Beweise für ihre Wirksamkeit sind unsicher.

## Ziele

Ziel dieses Reviews war, den Nutzen und die Risiken verschiedener Antioxidantien zu vergleichen, die bei männlicher Subfertilität eingesetzt werden.

## Suchmethodik

Das Studienregister der Cochrane Gynaecology and Fertility (CGF)-Gruppe, CENTRAL, MEDLINE, Embase, PsycINFO, AMED und zwei Studienregister wurden am 15. Februar 2021 durchsucht, außerdem wurden Referenzen überprüft und Experten auf dem Gebiet kontaktiert, um weitere Studien zu ermitteln.

## Auswahlkriterien

Wir schlossen randomisierte kontrollierte Studien (RCT) ein, die eine beliebige Art, Dosis oder Kombination von oralen Antioxidantien mit Placebo, keiner Behandlung oder einer Behandlung mit einem anderen Antioxidans bei subfertilen Männern eines Paares, die eine Reproduktionsklinik besuchen, verglichen. Wir schlossen Studien aus, in denen Antioxidantien mit Fruchtbarkeitsmedikamenten allein verglichen wurden sowie Studien, die Männer mit idiopathischer Unfruchtbarkeit und normalen Samenparametern oder fruchtbare Männer einschlossen, die eine Fruchtbarkeitsklinik wegen der Unfruchtbarkeit ihrer Partnerin aufsuchten.

## Datensammlung und Analyse

Wir haben die von Cochrane empfohlenen methodischen Standardverfahren angewandt. Das primäre Untersuchungsergebnis war die Lebendgeburt. Klinische Schwangerschaft, unerwünschte Ereignisse und Spermienparameter waren sekundäre Endpunkte.

## Hauptergebnisse

Insgesamt wurden 90 Studien mit einer Gesamtpopulation von 10.303 subfertilen Männern im Alter zwischen 18 und 65 Jahren einbezogen, die Teil eines Paares waren, das an eine Fruchtbarkeitsklinik überwiesen wurde und von denen sich einige einer medizinisch unterstützten Reproduktion (MAR) unterzogen. Die Studienautoren verglichen 20 verschiedene Antioxidantien. Der Evidenzgrad war von niedriger bis sehr niedriger Vertrauenswürdigkeit. Hier war die vorrangige Einschränkung, dass von den 67 in die Metaanalyse inkludierten Studien nur 20 die Endpunkte Schwangerschaft und Lebendgeburt berichteten. Die Evidenz war aktuell zum Zeitpunkt Februar 2021.

## Lebendgeburten

Antioxidantien können zu einer erhöhten Lebendgeburtenrate führen (Odds Ratio [OR] 1,43; 95 %-Konfidenzintervall [KI] 1,07 bis 1,91; *p* = 0,02; 12 RCT; 1283 Männer; I^2^ = 44 %; sehr niedrige Vertrauenswürdigkeit der Evidenz). Die Ergebnisse der Studien, die zur Analyse der Lebendgeburtenrate beigetragen haben, deuten darauf hin, dass die Chance auf eine Lebendgeburt nach der Einnahme von Placebo oder keiner Behandlung bei 16 % liegt, während die Chance nach der Einnahme von Antioxidantien auf 17–27 % geschätzt wird. Dieses Ergebnis basierte jedoch auf nur 246 Lebendgeburten von 1283 Paaren in 12 kleinen oder mittleren Studien. Wenn Studien mit hohem Verzerrungsrisiko aus der Analyse entfernt wurden, gab es keinen Hinweis auf eine erhöhte Lebendgeburt (Peto OR 1,22; 95 %-KI 0,85 bis 1,75; 827 Männer; 8 RCT; *p* = 0,27; I^2^ = 32 %).

## Klinische Schwangerschaftsrate

Antioxidantien können im Vergleich zu Placebo oder keiner Behandlung zu einer erhöhten klinischen Schwangerschaftsrate führen (OR 1,89; 95 %-KI 1,45 bis 2,47; *p* < 0,00001; 20 RCT; 1706 Männer; I^2^ = 3 %; niedrige Vertrauenswürdigkeit der Evidenz). Daraus ergibt sich, dass in den Studien, die zur Analyse der klinischen Schwangerschaft beigetragen haben, die Wahrscheinlichkeit einer klinischen Schwangerschaft nach der Einnahme von Placebo oder keiner Behandlung auf 15 % geschätzt wird, während die Wahrscheinlichkeit nach der Einnahme von Antioxidantien zwischen 20 und 30 % liegt. Dieses Ergebnis stützt sich auf 327 klinische Schwangerschaften von 1706 Paaren in 20 kleinen Studien.

## Unerwünschte Ereignisse

Fehlgeburten: Lediglich 6 Studien berichteten von diesem Endpunkt, darüber hinaus war die Ereigniszahl insgesamt sehr niedrig. Es wurden keine Hinweise auf einen Unterschied in der Abortrate zwischen der Gruppe mit Antioxidantien und der Gruppe mit Placebo oder ohne Behandlung gefunden (OR 1,46; 95 %-KI 0,75 bis 2,83; *p* = 0,27; 6 RCT; 664 Männer; I^2^ = 35 %; sehr niedrige Vertrauenswürdigkeit der Evidenz). Die Ergebnisse deuten darauf hin, dass in einer Population subfertiler Paare mit männlicher Unfruchtbarkeit und einer erwarteten Fehlgeburtenrate von 5 % das Risiko einer Fehlgeburt nach der Einnahme eines Antioxidans zwischen 4 und 13 % liegen würde.

## Gastrointestinale unerwünschte Ereignisse

Antioxidantien können im Vergleich zu Placebo oder keiner Behandlung zu einer Zunahme von leichten Magen-Darm-Beschwerden führen (OR 2,70; 95 %-KI 1,46 bis 4,99; *p* = 0,002; 16 RCT; 1355 Männer; I^2^ = 40 %; niedrige Vertrauenswürdigkeit der Evidenz). Daraus ergibt sich, dass, wenn die Wahrscheinlichkeit von Magen-Darm-Beschwerden nach einem Placebo oder keiner Behandlung mit 2 % angenommen wird, die Wahrscheinlichkeit nach der Verwendung von Antioxidantien schätzungsweise zwischen 2 und 7 % liegt. Allerdings beruhte dieses Ergebnis auf einer niedrigen Ereignisrate von 46 von 1355 Männern in 16 kleinen oder mittelgroßen Studien, und die Sicherheit der Beweise wurde als gering und die Heterogenität als hoch eingestuft. Die Autoren konnten keine Schlussfolgerungen aus jenen Studien ziehen, welche verschiedene Antioxidantien miteinander verglichen, da eine zu geringe Anzahl dieser Studien dieselben Interventionen verglichen.

## Schlussfolgerung der Autoren

In dieser Übersichtsarbeit gibt es Hinweise mit sehr geringer Vertrauenswürdigkeit der Evidenz aus 12 kleinen oder mittelgroßen randomisierten kontrollierten Studien, die darauf hindeuten, dass eine Supplementierung mit Antioxidantien bei subfertilen Männern die Lebendgeburtenrate bei Paaren, die Fertilitätskliniken aufsuchen, verbessern kann. Evidenz von niedriger Vertrauenswürdigkeit deutet darauf hin, dass die klinischen Schwangerschaftsraten möglicherweise steigen könnten. Es gibt keine Hinweise auf ein erhöhtes Risiko für Fehlgeburten, allerdings können Antioxidantien zu leichteren Magen-Darm-Beschwerden führen, die auf sehr unsicheren Erkenntnissen beruhen. Subfertile Paare sollten darauf hingewiesen werden, dass die aktuelle Evidenz hierzu insgesamt unschlüssig ist, hauptsächlich wegen eines signifikanten Risikos für Bias aufgrund mangelnder Darstellung der Randomisierungsmethoden, fehlender Aufzeichnung der Endpunkte „Lebendgeburtenrate“ und „Schwangerschaftsrate“, oftmals unklarer oder hoher Studienausstiegsraten sowie Ungenauigkeiten wegen niedriger Ereignisraten und insgesamt niedriger Fallzahlen.

Weitere große, gut konzipierte, randomisierte, placebokontrollierte Studien, die unfruchtbare Männer untersuchen und über Schwangerschaft und Lebendgeburten berichten, sind noch erforderlich, um die genaue Rolle von Antioxidantien zu klären.

## Kommentar

Paaren mit unerfülltem Kinderwunsch, wobei auf männlicher Seite keine spezifische Ursache der Unfruchtbarkeit gefunden wurde, werden oft künstliche Reproduktionsverfahren angeboten. Diese sind kostenintensiv und bieten keine ursächliche Behandlung an. Hier wirbt eine immer größer werdende Industrie mit Nahrungsergänzungsmitteln wie Antioxidantien. Da diese nicht den klassischen Zulassungsprozess als Medikament durchlaufen müssen, stehen auch weniger Daten zur Interpretation ihrer Wirksamkeit und Sicherheit zur Verfügung. In diesem Review wurden relevante Studien zu dieser Fragestellung zusammengefasst.

Der objektivste Parameter in der Untersuchung zur Wirksamkeit einer Behandlung der Kinderlosigkeit ist die Lebendgeburt eines Kindes. Leider wurde hier nur in 12 der 90 inkludierten Studien darüber berichtet, was die Aussagekraft sehr niedrig macht. In 4 dieser 12 Studien besteht zudem ein hohes Risiko für Verzerrung der Daten (Bias). Werden diese vier aus der Analyse weggelassen, so findet sich überhaupt kein Effekt von Antioxidantien auf die Lebendgeburtenrate. Anderes lassen die Daten bei der Schwangerschaftsrate vermuten. Hier bleibt ein kleiner Effekt auch bestehen, wenn nur Studien mit geringem Verzerrungsrisiko in die Analyse inkludiert werden.

Großes Interesse besteht auch zur Rate an unerwünschten Wirkungen bei Antioxidantien. Allen voran wurde hier die Fehlgeburtenrate untersucht. Leider fanden sich auch zu dieser Fragestellung nur in sechs Studien Aussagen, mit einer Ereigniszahl von lediglich 39, darüber hinaus war das Risiko auf Bias auch hier erheblich hoch. Objektivierbare Parameter, welche auf die Spermatogenese rückschließen lassen könnten, wie das Spermiogramm oder der DNA-Fragmentationsindex, konnten nicht gesammelt beurteilt werden, da fast jede Studie anders darüber berichtete. Ähnliches trifft auf die wenigen Vergleichsstudien mehrerer Antioxidantien zu.

Diese Übersichtsarbeit kommt bei den meisten vergleichbaren Fragestellungen zu ähnlichen Antworten wie rezente vorangegangene Reviews [1–4]. Eine weitere Cochrane-Übersichtsarbeit zur selben Fragestellung bei subfertilen Frauen sowie eine systematische Übersichtsarbeit mit Metaanalyse bei subfertilen Paaren, wo die Einnahme von Antioxidantien bei beiden Partnern untersucht wurde, zeigte einen geringen Vorteil für Antioxidantien bei Schwangerschaftsrate und Lebendgeburten, jedoch war auch hier die Aussagekraft und Literatur begrenzt [1, 5].

Zusammenfassend kann gesagt werden, dass subfertile Männer mit unerfülltem Kinderwunsch auf Basis dieser Studie beraten werden können, Antioxidantien zu versuchen, wenn sie invasivere Methoden ablehnen. Es scheinen keine schwerwiegenden Risiken bei einer Einnahme zu erwarten sein. Jedoch muss klar kommuniziert werden, dass die Evidenzlage für Wirkung und Risiko gering ist. Ebenso kann keine Empfehlung für bestimmte Antioxidantien oder eine bestimmte Dosierung ausgesprochen werden, da auch hierfür die Daten unzureichend waren.

### Limitationen

Insgesamt wurde die Vertrauenswürdigkeit der Evidenz als sehr gering bis gering eingeschätzt. Die vorrangigen Einschränkungen der diskutierten Übersichtsarbeit ergeben sich aus der geringen Anzahl der berichteten Endpunkte „Lebendgeburten“ und „Schwangerschaftsrate“ – über diese wurden lediglich in 12 bzw. 20 der 67 in die Metaanalyse eingeschlossenen Studien berichtet. Auch die Qualität der einzelnen eingeschlossenen Studien war limitiert – Studienmethoden wurden ungenau berichtet, Ungenauigkeiten, kleine Studienpopulation, „reporting bias“ und fehlende Daten waren hier vorrangig.

### Ausblick

Die eingeschränkte Aussagekraft der vorliegenden Übersichtsarbeit resultiert zu großen Teilen aus relevanten methodischen Schwächen der vorliegenden Studien sowie aus der kleinen und heterogenen Studienlandschaft zu diesem Thema. Hier sind große, qualitativ hochwertige Studien mit adäquater statistischer Power und homogener Endpunktgestaltung notwendig, um eine bessere Aussage zur Wirksamkeit und Wahrscheinlichkeit unerwünschter Wirkungen zu gestatten. Von der Industrie ist so ein kostspieliger und aufwändiger Vorstoß derzeit nicht zu erwarten, da die Marktlage dies wohl nicht erforderlich macht.
